# Immunoprophylaxis with MV140 Is Effective in the Reduction of Urinary Tract Infections—A Prospective Real-Life Study

**DOI:** 10.3390/vaccines12121426

**Published:** 2024-12-18

**Authors:** Filipe Abadesso Lopes, Miguel Miranda, André Ye, Joana Rodrigues, Paulo Pé-Leve, José Palma Reis, Ricardo Pereira e Silva

**Affiliations:** 1 Urology Department, Hospital de Santa Maria, 1649-028 Lisbon, Portugal; msmmmiranda@gmail.com (M.M.); jinandreye@gmail.com (A.Y.); joanaerodrigues95@gmail.com (J.R.); paulo.peleve@gmail.com (P.P.-L.); jpalmareis@campus.ul.pt (J.P.R.); ricardomanuelsilva7@gmail.com (R.P.e.S.); 2 Clínica Longeva, 1050-111 Lisboa, Portugal; 3 Faculdade de Medicina, Universidade de Lisboa, 1649-028 Lisbon, Portugal

**Keywords:** urinary tract infections, immunotherapy, vaccines

## Abstract

Background/Objectives: Urinary tract infections (UTI) represent a highly frequent and debilitating disease. Immunoactive prophylaxis, such as the polyvalent bacterial whole-cell-based sublingual vaccine MV140, have been developed to avoid antibiotic use. However, the effectiveness of this tool in the Portuguese population is still unknown. This study aims at assessing the effectiveness of treatment with MV140 in a cohort of Portuguese patients presenting with recurrent UTIs. Methods: Prospective observational real-life study of 125 patients with complicated and uncomplicated recurrent UTIs treated with MV140. The primary outcome was a reduction in frequency and severity of UTIs after a follow-up of 12 months. Overall satisfaction, adverse events, and assessment of the effectiveness of MV140 in subgroups of patients with specific risk factors for UTIs were secondary outcomes. Results: In the 12 months after treatment outset, 38% of patients were UTI-free, 34% reported 1 or 2 UTI episodes, and the remaining 28% presented 3 or more UTIs, corresponding to a mean reduction of 3.20 (2.87–3.53, 95% C.I.; *p* < 0.001) UTI episodes per year per patient. The effectiveness of MV140 was the same regardless of sex, BMI, regular sexual activity, hypertension, diabetes mellitus, depression, paraplegia, performance of intermittent self-catheterization, indwelling bladder catheter, or previous use of other UTI-preventing vaccines. We observed a higher effectiveness in post-menopausal women compared to pre-menopausal (74.7% vs. 59.4%, respectively, *p* = 0.029). A total of 73% of patients reported a reduction in symptom severity or days of disease, and the mean global satisfaction was 7.52/10. Conclusions: MV140 demonstrated to be effective in the reduction rate of recurrent UTIs in a cohort of adult Portuguese patients.

## 1. Introduction

The urinary tract infection (UTI) is the most frequent bacterial infection in humans. It is defined as the finding of significant bacteriuria accompanied by urinary symptoms, such as increased bladder sensation, urgency, frequency, dysuria, urinary incontinence, and/or pain [1]. UTIs are also the most common nosocomial infections, associated with severe health-related costs. The high incidence of UTIs and the use of antibiotics contributes to the increasingly concerning issue of antibiotic resistance.

Recurrent UTIs are defined as the diagnosis of three or more symptomatic and medically diagnosed UTIs over a period of 12 months or two UTIs in 6 months [1]. This is a common condition, especially in women, with an estimated prevalence of 0.1% in the female population aged 18–64 years in developed countries. About 80% of UTIs occur in women, and 20–30% have a recurrence within a year [2].

In the presence of recurrent UTIs, the use of long-term prophylactic antibiotics is common. Alternative therapies, such as cranberry products, D-mannose, herbal therapy with Canephron N, vitamin D, and probiotics, among others, have been widely tested, with unsatisfactory results [3]. Behavioral modifications, although commonly recommended, have not been supported by robust evidence [4]. Therefore, antibiotic prophylaxis is still mainstay for UTI prevention in severe cases [5].

In the bladder mucosa, the presence of a barrier formed by the urothelium, as well as neutrophils, macrophages, dendritic cells, natural killer cells, and mast cells, participates in the innate immune system. These cells contribute decisively to host defense by direct fighting of pathogens as well as by activation of the adaptive immune system. More recently, the mucosal immune system has been the scope of intense research, both aimed at the induction of a response against pathogens and at immunomodulation for the treatment of autoimmune diseases [6]. The mucosa-associated lymphoid tissue (MALT) represents an important component of the immune system, often responsible for the initiation of immune responses. Small clusters of immature lymphocytes and dendritic cells, among others, function as antigen-presenting cells, which promote the engagement of both humoral and cellular immune pathways. The sensitized B and T cells leave the site of the initial antigen presentation and migrate via blood or lymphatic vessels, promoting effector cells in the whole body and the differentiation of memory cells. This allows for a much more effective response to pathogens, via mechanisms similar to those involved in immunization via vaccines [7].

MV140 (Uromune^®^) is a sublingual spray manufactured by Inmunotek (Madrid, Spain). It consists of a suspension of whole-cell heat-inactivated bacteria and includes four selected bacterial strains—25% *Escherichia coli* (V121), 25% *Klebsiella pneumoniae* (V113), 25% *Enterococcus faecalis* (V125), and 25% *Proteus vulgaris* (V127)—in a suspension of 300 Formazin Turbidity Units of inactivated whole bacteria. Together, these strains account for around 85% of bacterial isolations in urine in UTIs in Portugal [8]. This mucosal immunotherapy is approved for the prevention of recurrent UTIs caused by these pathogens. The solution is administered once daily for 3 months (two sublingual sprays). Recent Spanish and British trials report an effectiveness of 50–90% in the prevention of UTIs at 12 months, with virtually no side effects [9,10,11]. These are very encouraging numbers given the low effectiveness and frequent side effects related to antibiotic prophylaxis.

Given the variation in the prevalence of bacterial pathogens in different regions, it may be suggested that treatment efficacy may also vary [8,12]. The main objective of this real-life study was to assess the effectiveness of MV140 in the prevention of UTIs in the Portuguese population. Furthermore, we assessed whether any sub-group of patients responded differently (e.g., differences according to sex, age, pre/post-menopause status, etc.). Finally, we aimed at reporting any side effects of MV140, as well as the global satisfaction with the treatment.

## 2. Materials and Methods

We conducted a prospective observational real-life study reporting the frequency and severity of UTIs in a cohort of patients suffering from recurrent UTIs treated with MV140. We recruited 125 consecutive patients treated at two centers in Portugal after prescription from their attending urologist. Inclusion criteria were as follows: (1) adult patients (>18 years old); (2) at least 3 positive urine culture UTIs in the preceding 12 months; (3) ability to understand and sign the informed consent form. Exclusion criteria were history of genitourinary tumors, urinary lithiasis, immunodeficiency, pregnancy, and simultaneous use of any other immunoactive prophylaxis or preventive antibiotics. At baseline, before starting the treatment, patients were asked to fill an informed consent form and the pre-treatment questionnaire, which included demographic and clinical history data. The second moment of assessment was at 12 months post-treatment, when a clinician contacted the subjects via phone to assess effectiveness and reports of any side effects of MV140, as well as overall satisfaction on a scale of 1–10 (with 1 corresponding to a low satisfaction and 10 to a high satisfaction). In case of UTI symptoms during the 12-month study period, patients were asked to perform urine cultures before any antibiotic treatment, and a UTI episode was considered as a combination of symptoms (dysuria, pollakiuria, fever, and/or urine odor) and positive urine culture (urine culture with >10^5^ CFU/mL).

This study was approved by the local Ethics Committee and was performed in accordance with the Declaration of Helsinki as revised in Fortaleza, Brazil, 2013. All patient data were anonymized.

Statistical analysis was performed using SPSS v29. A minimum sample size of 104 had been calculated prior to first patient inclusion, applying Cochran’s formula to detect a difference of 1 UTI episode at a significance level of 0.05 for the primary endpoint. Associations between the number of UTIs were tested using Wilcoxon test. Normality (using Kolmogorov–Smirnov tests) and variance homogeneity were calculated, and the associations of continuous variables with categorical or dichotomous variables were tested using Wilcoxon and Mann–Whitney tests, as appropriate. Statistical significance was set at *p* < 0.05.

## 3. Results

### 3.1. Study Group

We included 125 consecutive patients in the study. Of these, five were excluded for not completing the treatment, two due to the beginning of concomitant prophylactic antibiotics, and another ten patients as a consequence of unavailability of data, namely impossibility of post-treatment contact. The final study population was comprised of 108 patients, 84 women and 24 men, with a mean age of 54.9 ± 17.8 (22–88) years at the beginning of therapy. In the 12 months preceding treatment with MV140, each patient had experienced a mean of 4.68 ± 1.39 culture-positive UTIs. Further characteristics of the studied population are shown in Table 1.

### 3.2. Overall Effectiveness

**Figure 1:**
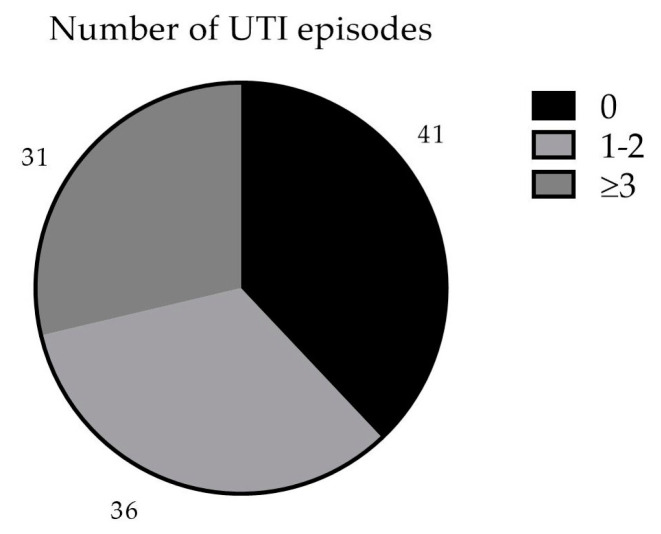
Number of UTI episodes in the first 12 months after MV140.

In the 12 months following the initiation of MV140, 41 patients (38%) were UTI-free, 36 (34%) reported one or two UTI episodes, and the remaining 31 patients (28%) presented three or more UTIs (Figure 1). We observed a mean reduction of 3.20 (2.87–3.53, 95% C.I.; *p* < 0.001) UTI episodes per year per patient, which means a mean reduction of 67.0% in UTI episodes. Only 8 patients reported an equal or higher number of UTI episodes.

### 3.3. Differential Effectiveness

There was no statistical difference between women and men regarding the reduction of episodes of UTI (reduction of 66.2% and 73.2%, respectively; *p* = 0.129). Regarding age, we observed a trend for greater reduction in UTIs in older patients (r^2^ = 0.029, *p* = 0.078), which was statistically significant for women (r^2^ = 0.071, *p* = 0.014) but not for men (r^2^ = 0.002, *p* = 0.852). When evaluating whether patient features had an impact on UTI reduction with MV140, there was no statistically significant variation regarding BMI, regular sexual activity, smoking habits, arterial hypertension, diabetes mellitus, pelvic surgery history, depression, paraplegia, indwelling bladder catheter, performing intermittent self-catheterization (ISC), or previous UTI prevention vaccine (Table 2). The analysis of the female population revealed a statistically significant difference between pre- and post-menopausal women, with the latter experiencing a greater reduction in UTI episodes (59.4% and 74.7%, respectively, *p* = 0.029). The analysis of number of births, hysterectomy, or incontinence/prolapse surgery history revealed no differences between subgroups (Table 3).

### 3.4. Secondary Outcomes—Symptom Severity, Side Effects and Global Satisfaction

Regarding symptom severity, of the 67 patients that reported UTIs in the year following the start of immunotherapy, 49 (73%) reported a reduction in severity and/or duration of symptoms, while the other 18 patients (27%) felt no difference. No patient reported more severe symptoms after treatment with MV140.

During the telephone contact 12 months after treatment with MV140, patients were also questioned about any possible side effects felt during/after treatment. These were reported by five patients, namely fatigue (2), nausea (2), and abdominal discomfort (1). All adverse events were considered mild by the patients, were self-limited, and did not lead to treatment discontinuation. Of note, a few patients reported a dislike for the taste of the spray, which was not considered an adverse event.

Finally, patients were asked about their overall satisfaction with the treatment on a scale of 1–10. The mean score was 7.52 ± 1.95, with 58% of patients rating their satisfaction as ≥8.

## 4. Discussion

We believe that this study with real-world evidence may contribute to the use of a new tool to prevent recurrent UTIs. Since commensal bacterial strains and infectious pathogens are highly geographically variable, it is important to assess whether antibiotic or non-antibiotic treatments are effective in the prevention of recurrent UTIs in each population.

Our findings suggest that treatment with the sublingual vaccine MV140 is effective in the prevention of UTIs in a cohort of Portuguese patients with recurrent UTIs. In fact, 93% of patients reported a reduction in UTI episodes in the year following treatment, and 38% reported no episodes of UTI. Moreover, the severity of symptoms seems to be reduced by the therapy, with 73% of patients reporting a reduction in either symptom severity (e.g., pain of hematuria) or duration (in days). This reduction in symptom severity was not previously reported, but we believe that this fact contributed decisively to the high satisfaction of patients (7.52/10).

Previously published literature reported a UTI-free ratio of 50–90% at 12 months with MV140 [9,10,11,13,14,15,16]. In the present study, we found a slightly shorter benefit, with 38% of patients being UTI-free at 12 months post-treatment. This could be explained by differences in pathogen distribution but also by the fact that this was a real-life study, and hence the studied population included patients with complicated UTIs (such as men, paraplegic patients, and patients performing ISC or with indwelling catheters). Even though there was not a statistically significant difference of effectiveness in these subgroups, a slight shift in the UTI-free rate may have happened.

To our knowledge, only two studies have been conducted regarding MV140 use in men, with less optimal effectiveness in this population (30% UTI-free rate at 6 months) [9,11]. Conversely, in the present study, no significant differences in effectiveness were found according to sex. Despite differences in commensal flora, the most common pathogens responsible for UTIs are similar between men and women [12]. Of note, the effectiveness of the vaccine was higher in post-menopausal women than in pre-menopausal women. Previously published studies report conflicting results, with some cohorts presenting the same association [9] and others reporting the opposite [10]. In our cohort, the explanation for this phenomenon is not clear, although simultaneous treatment of other comorbidities such as vulvovaginal atrophy or urinary incontinence may have been initiated after the same visit in which MV140 was prescribed, as the study was conducted in a real-life setting. This could have played a role in the reduction of UTIs mostly in post-menopausal women.

This study is also the first to specifically assess the effectiveness of MV140 in the prevention of complicated UTIs, namely in patients with neurogenic bladder, indwelling bladder catheters, or performing ISC. In all these subgroups, we found no statistically significant variability in effectiveness, suggesting that these patients may benefit from this vaccine, although this study was not specifically designed to study patients with neurogenic lower urinary tract dysfunction, and therefore the sample size in this subgroup was very small.

Possible limitations of this study include the fact that the outcomes were obtained mainly through patient-reported data and relies on the comparison of UTI experience for each patient, and therefore recall bias may be present in both the pre- and post-treatment questionnaire. However, when patients reported exacerbation of symptoms in possible relation with UTI, urinalysis and urine cultures were obtained to rule out infection. The sample size may also have been insufficient, especially in certain subgroups, and the “real-world” design of the study (without a placebo control group) are also clear limitations. Furthermore, other measures such as behavioral changes or vaginal estrogen, which were not exclusion criteria, could account for some improvement in UTI frequency. Lastly, the second questionnaire was performed 12 months after the beginning of treatment, which may have resulted in missing data and the consequent exclusion of patients from analysis, as reported above.

## 5. Conclusions

In this prospective real-life study, we found that MV140 was effective in the reduction rate of recurrent UTIs in a cohort of Portuguese male and female patients. Sublingual vaccines, such as MV140, may, in the future, be the cornerstone for prevention, even in patients with complicated UTIs, if future placebo-controlled research is able to reproduce the results of this study.

## Figures and Tables

**Table 1:** Population characterization.

Study Population	n = 108 (100%)
Regular sexual activity	69 (64%)
Obesity (BMI > 30)	11 (10%)
Smoking habits	19 (18%)
Arterial hypertension	27 (25%)
*Diabetes mellitus*	8 (7%)
Pelvic surgery history	16 (15%)
Depression	22 (20%)
Stroke	1 (1%)
Dementia	2 (2%)
Paraplegia	4 (4%)
Indwelling bladder catheter	4 (4%)
Performing ISC	9 (8%)
Previous UTI prevention oral vaccine	50 (46%)
Female Patients	n = 84 (100%)
Post-menopause	42 (50%)
Births	56 (67%)
Eutocic	34 (40%)
Forceps/vacuum use	9 (11%)
Cesarian section	26 (31%)
Hysterectomy history	18 (21%)
Prolapse/incontinence surgery history	7 (8%)
Male Patients	n = 24 (100%)
BPO medication	6 (25%)
BPO surgery history	4 (17%)

BMI—body mass index; ISC—intermittent self-catheterization, UTI—urinary tract infection; BPO—benign prostatic obstruction.

**Table 2:** Population differential analysis.

	Mean No. UTIs Before MV140	Mean No. UTIs After MV140	Reduction Ratio	*p*-Value
**Study Population (108)**	**4.79**	**1.58**	**67.0%**	<0.001 *
Regular sexual activity				0.150 ^+^
No (39)	4.67	1.26	75.6%	
Yes (69)	4.85	1.77	65.3%	
Smoking habits				0.118 ^+^
No (89)	4.73	1.46	71.0%	
Yes (19)	5.00	2.26	58.3%	
Arterial hypertension				0.122 ^+^
No (81)	4.87	1.70	67.1%	
Yes (27)	4.50	1.17	75.8%	
Diabetes mellitus				0.262 ^+^
No (100)	4.83	1.46	72.0%	
Yes (8)	4.33	2.89	58.8%	
Pelvic surgery history				0.159 ^+^
No (92)	4.85	1.70	66.8%	
Yes (16)	4.63	1.11	78.6%	
Depression				0.553 ^+^
No (86)	4.77	1.61	68.3%	
Yes (22)	4.83	1.48	72.0%	
Paraplegia				0.488 ^+^
No (104)	4.77	1.56	69.6%	
Yes (4)	5.25	2.25	55.8%	
Indwelling bladder catheter				0.643 ^+^
No (104)	4.76	1.54	69.5%	
Yes (4)	5.20	2.40	59.3%	
Performing ISC				0.302 ^+^
No (99)	4.81	1.53	69.6%	
Yes (9)	4.78	2.33	59.5%	
Previous UTI prevention vaccine				0.427 ^+^
No (58)	4.51	1.48	70.3%	
Yes (50)	5.08	1.72	67.1%	

*—Wilcoxon test; ^+^—Mann–Whitney test; ISC—intermittent self-catheterization, UTI—urinary tract infection.

**Table 3:** Female and male population analysis.

	Mean No. UTIs Before MV140	Mean No. UTIs After MV140	Reduction Ratio	*p*-Value
**Female Patients (84)**	**4.76**	**1.67**	**66.2%**	<0.001 *
Menopause				0.029 ^+^
Pre-menopause (42)	4.83	2.00	59.4%	
Post-menopause (42)	4.69	1.33	74.7%	
Births				0.708 ^+^
No (28)	4.79	1.54	68.9%	
Yes (56)	4.75	1.73	66.1%	
Hysterectomy history				0.865 ^+^
No (66)	4.77	1.71	66.3%	
Yes (18)	4.72	1.61	68.0%	
Prolapse/incontinence surgery history				0.652 ^+^
No (77)	4.78	1.70	66.4%	
Yes (7)	4.00	1.33	73.3%	
Male Patients (24)	4.89	1.27	73.3%	0.019 *

*—Wilcoxon test; ^+^—Mann–Whitney test.

## Data Availability

Data supporting the reported results may be obtained through contact with the corresponding author.
